# Flow-Based Chemiluminescence Microarrays as Screening Platform for Affinity Binders to Capture and Elute Bacteria

**DOI:** 10.3390/s22228606

**Published:** 2022-11-08

**Authors:** Julia Neumair, Martin Elsner, Michael Seidel

**Affiliations:** TUM School of Natural Sciences, Department of Chemistry, Technical University of Munich, 85748 Garching, Germany

**Keywords:** pathogens, microarray, chemiluminescence, affinity

## Abstract

Affinity describes the non-covalent but selective interaction between an affinity binder (e.g., proteins, antibiotics, or antibodies) and its counterpart (e.g., bacteria). These affinity binders can serve to detect bacteria and respond to the need for selective concentration via affinity chromatography for trace analysis. By changing the pH value or salt and protein contents, affinity bindings can be reversed, and bacteria can be recovered for characterisation. Analytical microarrays use multiple affinity binders immobilised on the surface in a distinct pattern, which immensely reduces screening time for the discovery of superior binding motifs. Here, flow-based microarray systems can inform not only about binding, but also about desorption. In this work, we pioneer a screening assay for affinity binders against both gram-positive and negative bacteria based on an automated flow-based chemiluminescence (CL) microarray. Biotinylation of model organisms *E. coli* and *E. faecalis* enabled labelling with horseradish-peroxidase-coupled streptavidin, and detection with CL. Polymyxin B, an antibiotic against gram-negative bacteria, was found to bind both *E. coli* and *E. faecalis*. Simultaneous screening for desorption methods unexpectedly revealed methyl alpha-D-mannopyranoside as a promising buffer for desorption from Polymyxin B. This proof-of-principle study shows that our new platform greatly facilitates the screening of new affinity binders against bacteria, with promise for future automation.

## 1. Introduction

The rapid and sensitive detection of bacteria is crucial in many areas like diagnostics, water and food analytics. Whilst pathogenic bacteria can already cause health problems in low concentrations, their detection at these low concentrations may be difficult. To overcome this problem, an enrichment of bacteria is necessary. Hereby, various methods for enrichment, such as centrifugation [1] or filtration [2], have been brought forward. If more specific enrichment methods are required, affinity-based methods [3,4,5] hold great promise. Here, an affinity between the used affinity binders and the bacterial cell walls is utilised to capture the bacteria and, in this way, to remove them from the sample matrix. Subsequently, a direct detection [6] or a desorption from the separation matrix before detection [7] can be performed. Various groups, such as antibodies [8], lectins [9,10], or antibiotics [3,5] can serve as affinity binders.

Affinity binding is also used in the concept of microarrays. Microarrays are multi-analyte platforms in which different types of probes are immobilised on the microarray chip surface. Utilizing the affinity between bacterial cells and immobilised probes, bacteria can be identified and quantified [11,12,13,14,15,16]. Although some microarrays follow a microtiter plate format [17,18], most are chip-based. To simplify the assay workload, many microarray assays rely on a lateral flow [19,20] or are flow-based [12,21,22]. For the latter, the liquid reagents are transported over the microarray chip surface by using a pump, which also allows for automation. The multiplexing manner of microarrays is one of its major advantages, as it allows for the simultaneous detection of multiple analytes with only one measurement. However, the interaction of one analyte with multiple immobilised probes can also be investigated. The detection of bacterial cells on a microarray can either be performed label-free—for example, with electrochemical sensors [23,24,25]—or via labelling of the bacterial cells. Labelling—either direct labelling of the bacterial cells [26] or via a labelled second probe—is often performed using fluorescence [18] or chemiluminescence (CL) markers [14,27]. 

In this work, an existing automated-flow-based CL microarray platform [28] was used to establish the first assay to study the adsorption and desorption properties of affinity binders towards bacteria. Our aim was to pioneer a novel tool to greatly facilitate the screening for new affinity binders and their corresponding desorption buffers. To enable detection of bacteria using a CL microarray, the cells were biotinylated using biotin 3-sulfo-N-hydroxysuccinimide ester sodium salt (sNHS-biotin) which forms covalent bonds with free amino groups at the bacterial cell surface. 

Different types of affinity binders were chosen to interact with the model organisms *Escherichia coli* and *Enterococcus faecalis*. The antibiotic polypeptide Polymyxin B (PmB) is a cyclic lipopeptide possessing a fatty acid tail [29,30] and is mainly used against gram-negative bacteria. Its cationic properties allow interaction with phospholipids and lipopolysaccharide structures of the cell wall of gram-negative bacteria [30]. It was already used in the affinity filtration of *Escherichia coli* [3] as well as in the removal of endotoxins [31]. Lysozyme is an enzyme that is known for its bacteriolytic properties, mostly for gram-positive bacteria. Hereby, it disrupts the peptidoglycans that the cell wall is built of [32]. As a third affinity binder, the lectin Concanavalin A (ConA) was used, which has a high affinity towards sugar moieties, which are present on the bacterial cell walls. It was already used in biosensors and for the enrichment of bacteria [33,34,35]. Additionally, antibodies were used as immuno-affinity binders, and one antibody against all *O* and *K* antigenic serotypes of *E. coli* and one against *Enterococcus species* were chosen. 

In this proof-of-principle study, we could identify PmB as a highly promising affinity binder to the gram-negative bacterium *E. coli* and gram-positive bacterium *E. faecalis*. This was unexpected, as PmB is only used as an antibiotic against gram-negative bacteria. The multiplex manner of the screening platform allowed fast testing of various desorption reagents for all affinity binders at once. This facilitated the discovery of methyl alpha-D-mannopyranoside (MADM) as a new promising desorption reagent for PmB, although it was originally applied to the lectin ConA. 

## 2. Materials and Methods

### 2.1. Materials and Buffers

If not stated otherwise, chemicals were purchased from Sigma Aldrich (Darmstadt, Germany), a subsidiary of Merck, or Carl Roth (Karlsruhe, Germany). Streptavidin was purchased from IBA Lifesciences (Göttingen, Germany) and horseradish-peroxidase-labelled streptavidin (HRP-streptavidin) from Biozol (Eching, Germany). CL reagents (luminol and hydrogen peroxide) were purchased as the Elistar Supernova reagent kit from Cyanagen (Bologna, Italy). *E. coli* serotype O/K polyclonal antibody and *Enterococcus* polyclonal antibody were provided by Thermo Fisher Scientific (Waltham, MA, USA). Polycarbonate foils (Makrolon^®^ GP, 0.25 mm) were obtained from Modulor (Berlin, Germany). *E. coli* (DSM 1003) and *E. faecalis* (DSM 2570) were bought from the German Collection of Microorganisms and Cell Cultures (Braunschweig, Germany). Ultrapure water was used unless stated otherwise. Experiments with viable bacteria were performed in a laboratory with a biosafety level of 2. 

Phosphate-buffered saline (PBS, pH 7.4) was prepared using 70 mM K_2_HPO_4_, 10 mM KH_2_PO_4_ and 145 mM NaCl. As running buffer for the MCR-R, a 0.1% Tween^®^ 20 solution in PBS (PBS-T) was used. Carbonate buffer with pH 9.6 was prepared from 15 mM Na_2_CO_3_ and 35 mM NaHCO_3_; beef extract glycine buffer (BEG, pH 9.5) consisted of 505 mM glycine and 3% beef extract powder. 

### 2.2. Bacterial Cultivation 

*E. coli* (DSM 1003) and *E. faecalis* (DSM 2570) from cryo-cultures (−80 °C) were cultivated on tryptic soy agar plates overnight at 37 °C. For the preparation of stock suspensions, cells were harvested, washed two times by centrifuging (10 min, 4500 rpm, 4 °C), and resuspending the sample in PBS (pH 8). Cell concentrations were determined via photometric measurements on a NanoPhotometer from Implen (Munich, Germany). 

### 2.3. Biotinylation

The freshly prepared bacterial stock suspensions in PBS (pH 8) were diluted to a working concentration of 10^9^ cells mL^−1^, and biotin 3-sulfo-N-hydroxysuccinimide ester sodium salt (sNHS-biotin) was added to achieve an end concentration of 2 mg mL^−1^. The reaction mixture was incubated on ice at 100 rpm for 30 min. Afterwards, the cells were washed twice with 0.1 M glycine in PBS and once with PBS (10 min, 4500 rpm, 4 °C). Finally, the cells were resuspended in PBS, and the cell concentration was determined using OD measurements and culture. The biotinylated bacteria were stored at 4 °C. 

### 2.4. Production of Microarray Chips

**Figure 1:**
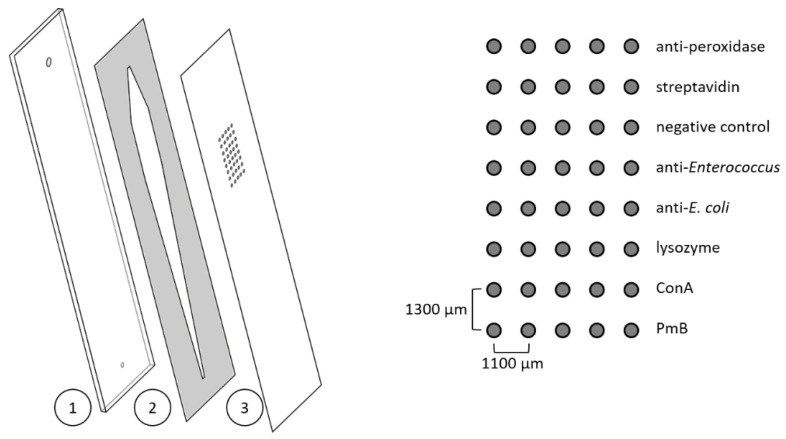
**Left**: scheme of a disassembled microarray chip consisting of a black polyoxymethylene carrier plate with in- and outlets (1); a double-sided adhesive with cut-out flow channel (2); and the polycarbonate sheet with immobilised reagents (3). On the **right**, the spotting scheme is shown.

Polycarbonate foils (0.25 mm) were used as a surface for the microarray chips and were prepared based on a protocol described elsewhere [36]. In short, foils were cut into a sheet of 3 × 3 chips in the size of 26 × 76 mm using the CE 6000–40 cutting plotter from Graphtec Corporation (Yokohama, Japan), coated with succinylated Jeffamine^®^ ED-2003 using a screen printer, and incubated at 95 °C for 2 h before washing and drying. Until further use, the sheets were stored at room temperature under reduced humidity. Affinity binders were immobilised in rows of five spots using the contact spotter BioOdyssey Calligrapher^®^ MiniArrayer from Bio-Rad (Hercules, USA). The distance between the spots of one row was 1100 µm, and the distance was 1300 µm between the spots of different rows (diameter of spots 150 µm). Spotting solutions contained 0.4 mg mL^−1^ 1-ethyl-3-(3-(dimethylamino)propyl)carbodiimide (EDC), 1.1 mg mL^−1^ *N*-hydroxysulfosuccinimide sodium salt (sulfo-NHS), and the reagents to be immobilised in PBS. End concentrations were 1 mg mL^−1^ for lysozyme, ConA, PmB, and the antibacterial antibodies. Polyclonal antiperoxidase antibody from rabbit (1:40 dilution) and streptavidin (1 mg mL^−1^) were used as positive controls; the negative control was the spotting solution without any further reagent. In Figure 1 (right side), the spotting scheme is shown. Spotting took place at 20 °C and 55% relative humidity, and the sheets were incubated overnight under the same conditions. Afterwards, the sheets were divided into individual pieces, and microarray chips were assembled using a black polyoxymethylene (POM) carrier plate with in- and outlets and a double-sided adhesive (thickness 140 µm) with a cut-out flow channel (56 µL, Figure 1, left). Microarray chips were stored at 4 °C until further use. 

### 2.5. Screening Assay

The whole assay—except for the desorption step—was automated on the Microarray Chip Reader—Research (MCR-R) built by GWK Präzisionstechnik (Munich, Germany). At the beginning of each measurement day, the device was set up by filling all tubes with running buffer and loading the used reagents (1% Casein in PBS for blocking, HRP-streptavidin diluted 1:2000 in running buffer, and the individual CL reagents (luminol and hydrogen peroxide)). The microarray chip holder on the MCR-R was heated to 35 °C. For every microarray chip, a darkframe picture was taken. For this, the microarray chip was inserted directly before measurement into the MCR-R, flushed with running buffer, and an image was recorded for 60 s without adding any reagent. 

The sample to be measured was injected into the sample port, and the measuring program was started. A total of 604 µL of the sample was first transported to the chip (50 µL s^−1^) and then passed over the chip (1 µL s^−1^) using a stopped flow consisting out of ten increments with an incubation time of 30 s each. After a washing step with running buffer (2000 µL, 150 µL s^−1^), the casein solution for blocking and the HRP-streptavidin solution were first transported to the chip (50 µL s^−1^) and then passed over the chip (both 600 µL, 5 and 2 µL s^−1^), followed each time by another washing step. Finally, the CL reagents (luminol and hydrogen peroxide) were mixed in a 1:1 ratio (both 200 µL) and injected into the chip (100 µL s^−1^), and an image was recorded immediately for 60 s. After an additional washing step (1000 µL, 200 µL s^−1^), the microarray chip was removed directly from the device, and desorption was performed manually. Using a pipette, 100 µL of desorption buffer was flushed through the chip. For experiments with an incubation step, the desorption buffer was incubated on the chip for 60 s and removed afterwards. The chip was then inserted back immediately into the device, and the previous steps were repeated, starting from the passing of the HRP-streptavidin. To avoid contamination between measurements, the tubes of the device were flushed during desorption (with a different chip) and after the second image acquisition (3 times 2500 mL, 500 µL s^−1^). The detailed measuring program on the MCR-R containing volumes and flow rates for every step is shown in Table 1. A schematic fluidic plan as well as the pathways for reagents are shown in Supplementary Materials Figure S1 and Table S1. 

### 2.6. Data Evaluation

The software of the MCR-R device automatically subtracts the darkframe CL signals from the CL signals obtained during the measurements. The resultant files were evaluated using the software MCR spotreader (Stefan Weißenberger, Munich, Germany). A grid was placed over the image, with one box per spot. The software calculated the CL signal for each box as the mean of the 10 brightest pixels. The output for every immobilised reagent is given as the mean of the corresponding row (5 spots), and spots which deviated by more than 10% were excluded. The CL signal is then normalised for every measured microarray chip by dividing the CL signal of the spotted affinity binders by the CL signal of the spotted negative control.

(1) $$\mathrm{normalised}\;\mathrm{CL}\;\mathrm{signal}\;\left(\mathrm{affinity}\;\mathrm{binder}\right)=\frac{\mathrm{mean}\;\mathrm{CL}\;\mathrm{signal}\;\left(\mathrm{spotted}\;\mathrm{affinity}\;\mathrm{binder}\right)}{\mathrm{mean}\;\mathrm{CL}\;\mathrm{signal}\;\left(\mathrm{spotted}\;\mathrm{negative}\;\mathrm{control}\right)}$$

The mean-normalised CL signal is given as the mean of the microarray chips measured with the corresponding standard deviation between experiments, whereas *n* is the number of experiments. 

Statistical analysis was performed in Excel using the Real Statistics Resource Pack software ((Release 7.6). Copyright (2013–2021) Charles Zaiontz. www.real-statistics.com, accessed on 20 October 2022). A Shapiro–Wilk test (α = 0.05) was performed to check for normal distribution of data, whereas variance homogeneity was investigated with a Levene’s test (results not shown, α = 0.05). Depending on the outcome of these two tests, either a one-factor analysis of variance (ANOVA, α = 0.05) followed by a Tukey HSD test (α = 0.05) or a Kruskal–Wallis test (α = 0.05) followed by a Conover test (α corrected with Bonferroni correction) was done. For effect sizes, Cohen’s *d* was calculated, classification was done according to Sawilowsky et al. [37].

## 3. Results and Discussion 

### 3.1. Assay Concept

**Figure 2:**
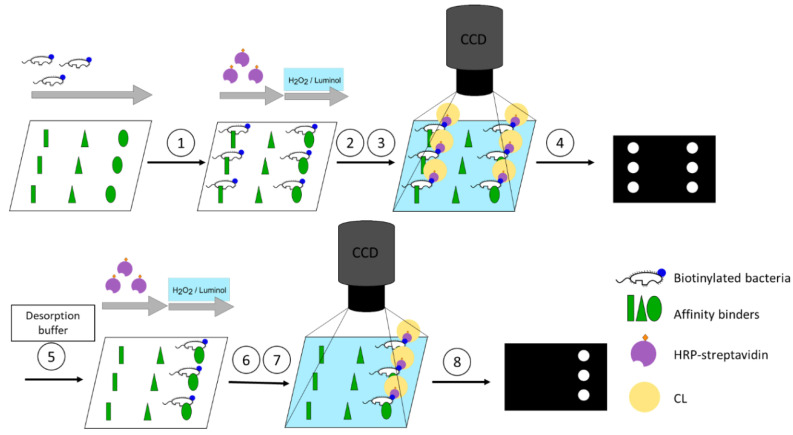
Concept of the screening assay. (1): Capture of the biotinylated bacteria through the affinity binders depending on the affinity. (2): Binding of the horseradish peroxidase (HRP)-labelled streptavidin. (3): Chemiluminescence (CL) reaction. (4): Image acquisition. (5): Desorption of bacteria by desorption buffer depending on reversing of affinity. (6): Binding of the HRP-streptavidin. (7): CL reaction. (8): Image acquisition.

The flow-based CL microarray assay was established on the MCR-R. On this platform, reagent addition as well as imaging are executed automatically. Volumes and flow rates of each reagent are controlled separately. Affinity binders are immobilised on the surface of the flow-through microarray chips. To start a measurement, the assembled microarray chip is inserted into the MCR-R. Samples containing biotinylated bacteria are incubated on the microarray chip in a stopped-flow manner in order to enhance the interaction time between the affinity binder and bacteria. For imaging, the HRP-streptavidin is flushed over the chip and binds to the biotin present at the bacteria’s cell wall. CL reagents luminol and H_2_O_2_ are mixed and flushed over the chip, after which the bound HRP-streptavidin catalyses the CL reaction and CL signals are recorded by a CCD camera installed in the MCR-R. For testing the desorption from the affinity binders, the microarray chip is removed and flushed by pipetting the desorption buffer into the chip and incubating depending on the desorption mode. The chip is inserted again for the second measurement starting from the HRP-streptavidin step (Figure 2).

### 3.2. Biotinylation of Bacteria

For detection of bound bacteria using CL via coupling with HRP-streptavidin, bacterial cells were biotinylated. As the biotinylation process consists of several washing steps, during which the cell suspension is centrifuged and the formed pellet is resuspended, cells could be lost or inactivated. The loss of total bacterial cells, or rather their recovery, was evaluated by photometric measurements, whereas their viability—or more specific, their culturability—was tested via culture. 

For *E. coli,* for the total cells, a recovery of 95 ± 16% (*n* = 10; *W*(9) = 0.97, *p* = 0.83) was found, indicating little to no cell loss. For the culturability, a recovery of 98 ± 51% (*n* = 7; *W*(6) = 0.98, *p* = 0.97) was found. An ANOVA showed no significant difference between these data (*F*(1,15) = 0.08, *p* = 0.77). 

At the same time, for *E. faecalis,* a recovery for total cells of only 70 ± 22% (*n* = 9; *W*(8) = 0.96, *p* = 0.84) and for culturable cells of 75 ± 29% (*n* = 7; *W*(6) = 0.95, *p* = 0.74) was found. Here again, no significant difference was found (*F*(1,15) = 0.16, *p* = 0.70). The recoveries for *E. coli* and *E. faecalis* total cells were significantly different (*F*(1,18) = 8.6, *p* = 0.01), which leads to the conclusion that the effect from the biotinylation process is diverse for different bacteria.

### 3.3. Assay Development

Before the assay can be used for the screening for binding and desorption of bacteria from affinity binders, the assay has to be established. For this, blank measurements (only PBS), control measurements with sNHS treated according to the biotinylation protocol, *E. coli* and *E. faecalis* without biotinylation, and measurements with the biotinylated bacteria were performed. For every affinity binder over the six different samples, a Kruskal–Wallis test was performed, and significant differences between the samples were found (PmB: *chi-square* (5) = 81.11, *p* = 4.90 × 10^−16^; lysozyme: *chi-square* (5) = 58.94, *p* = 2.01 × 10^−11^; anti-*E. coli*: *chi-square* (5) = 85.49, *p* = 5.95 × 10^−17^; anti-*Enterococcus*: *chi-square* (5) = 82.32, *p* = 2.74 × 10^−16^). Post-hoc Conover tests (corrected α = 0.003) were performed; the results therefore will be shown in the next relevant paragraphs.

**Figure 3:**
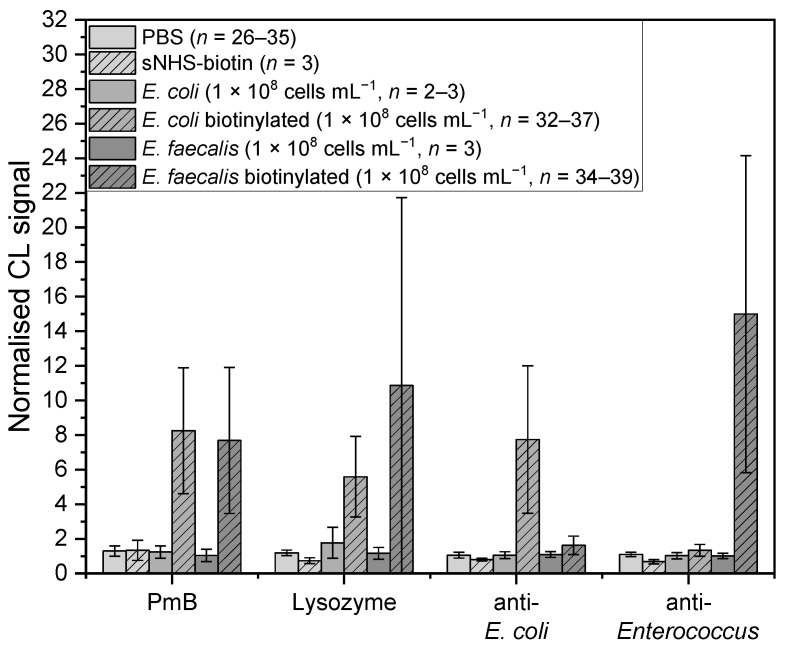
Normalised CL signals for the affinity binders Polymyxin B (PmB), lysozyme, *E. coli* serotype O/K polyclonal antibody (anti-*E. coli*) and *Enterococcus* polyclonal antibody (anti-*Enterococcus*). Control measurements for negative controls were conducted with PBS (*n* = 26–35, lightest grey), sNHS-biotin (*n* = 3, lightest grey shaded) and with bacteria without biotinylation (*n* = 2–3, medium and darkest grey). Positive control measurements were done with biotinylated bacteria (*n* = 32–39, medium and darkest grey shaded). Concentrations for measurements with bacteria were 1 × 10^8^ cells mL^−1^.

First, we checked if the obtained CL signals stemmed from bound bacteria or from any unspecific bindings. Testing for (unspecific) bindings between HRP-streptavidin and affinity binders was conducted by performing the assay without adding bacteria and measuring only with PBS (Figure 3, lightest grey, *n* = 26–35). For the affinity binders PmB (*n* = 35, *W*(34) = 0.97, *p* = 0.29), for lysozyme (*n* = 26, *W*(25) = 0.99, *p* = 0.99), and for both of the antibacterial antibodies (anti-*E. coli*: *n* = 35, *W*(34) = 0.98, *p* = 0.79; anti-*Enterococcus*: *n* = 33, *W*(32) = 0.98, *p* = 0.91), the mean-normalised CL signals were between 1.0 and 1.3, indicating little to no unspecific binding. For ConA, a specific interaction towards HRP was given [38], so a higher mean-normalised CL signal was expected. A mean-normalised CL signal of 37.9 ± 22.5 (*n* = 35, *W*(34) = 0.93, *p* = 0.03) confirmed these expectations, so we excluded ConA from further experiments.

Another effect on the CL signal could derive from any remaining free sNHS-biotin from the biotinylation process that could attach to the affinity binder and cause a signal. Therefore, the sNHS-biotin solution was treated the same way as for the biotinylation of bacteria. Normalised CL signals of immobilised streptavidin give information as to whether there is still sNHS-biotin left in the sample after washing or if it was completely removed. A Kruskal–Wallis test of the mean-normalised CL signal of the sNHS-biotin control with 9.0 ± 4.7 (*n* = 3) compared to 1.4 ± 0.4 (*n* = 36, *W*(35) = 0.95, *p* = 0.10) of the PBS blank shows that there is a significant difference between the data (*chi-square* (1) = 8.1, *p* = 0.004), which was confirmed with a Conover test (*t*(37) = 3.17, *p* = 0.003, *d* = 6.60). This indicates that some of the sNHS-biotin was indeed still left in the sample. Examining the mean-normalised CL signals (Figure 3, lightest grey, shaded, *n* = 3) for PmB (*t*(111) = 0.12, *p* = 0.91), lysozyme (*t*(95) = 1.53, *p* = 0.13) and the antibodies (anti-*E. coli*: *t*(114) = 2.20, *p* = 0.03; anti-*Enterococcus*: *t*(108) = 2.83, *p* = 0.006) with values between 0.7 and 1.3. However, no significant differences between the blank measurements and the sNHS measurements were found. To verify that biotinylation on the bacteria is necessary and that no unspecific binding between them and the HRP-streptavidin occurs, the assay was performed with non-biotinylated *E. coli* and *E. faecalis* (1 × 10^8^ cells mL^−1^) (Figure 3, middle and darkest grey, *n* = 2–3). Mean-normalised CL signals for PmB (*E. coli*: *n* = 2, *t*(111) = 0.14, *p* = 0.89, *E. faecalis*: *n* = 3, *t*(111) = 0.90, *p* = 0.37), lysozyme (*n* = 3, *E. coli*: *t*(95) = 1.86, *p* = 0.06, *E. faecalis*: *t*(95) = 0.31, *p* = 0.76), anti-*E. coli* (*n* = 3, *E. coli*: *t*(114) = 0.03, *p* = 0.98, *E. faecalis*: *t*(114) = 0.18, *p* = 0.86), and anti-*Enterococcus* (*n* = 3, *E. coli*: *t*(108) = 0.79, *p* = 0.43, *E. faecalis*: *t*(108) = 0.92, *p* = 0.36) were between 1.0 and 1.7. No significant difference from the blank measurements and therefore no unspecific binding was observed. 

Last, the assay was verified using the biotinylated bacteria (Figure 3, middle and darkest grey, shaded, 1 × 10^8^ cells mL^−1^). For *E. coli*, the two affinity binders PmB and lysozyme gave mean-normalised CL signals of 8.2 ± 3.6 (*n* = 36, *W*(35) = 0.98, *p* = 0.59) and 5.6 ± 2.3 (*n* = 32, *W*(31) = 0.97, *p* = 0.54), respectively, which showed a significant difference from the measurements in PBS (PmB: *t*(111) = 13.26, *p* = 1.21 × 10^−24^, *d* = 2.18; lysozyme: *t*(95) = 9.01, *p* = 2.15 × 10^−14^, *d* = 0.67), indicating an interaction with the bacterial cells. For the anti-*E. coli* antibody, the mean-normalised CL signal was 7.7 ± 4.3 (*n* = 37, *W*(36) = 0.96, *p* = 0.15, *t*(114) = 15.62, *p* = 4.18 × 10^−30^, *d* = 3.68), which also showed a significant difference from the blank measurements, whereas for the anti-*Enterococcus* antibody, it was 1.3 ± 0.3 (*n* = 37, *W*(36) = 0.98, *p* = 0.64, *t*(108) = 4.09, *p* = 8.39 × 10^−5^, *d* = 0.05), which is a significant difference from the PBS but with a very small effect size. This outcome was expected, as the aforementioned antibodies should bind or should not bind with *E. coli*, respectively. Observing the overall effect size, the anti-*E. coli* antibody had the greatest effect and is therefore considered the best affinity binder, followed by PmB. 

For *E. faecalis*, the two affinity binders PmB and lysozyme showed mean-normalised CL signals of 7.7 ± 4.2 (*n* = 38, *W*(37) = 0.91, *p* = 0.005, *t*(111) = 12.38, *p* = 1.25 × 10^−22^, *d* = 2.01), and 10.9 ± 10.9 (*n* = 34, *W*(33) = 0.83, *p* = 1.05 × 10^−4^, *t*(95) = 9.16, *p* = 1.03 × 10^−14^, *d* = 1.48), respectively, indicating significant differences to the blank measurement with PBS. The standard deviation for lysozyme was very high, suggesting a non-uniform interaction between the cells and the affinity binder. Additionally, the mean-normalised CL signals for the antibodies were as expected, with 1.6 ± 0.5 (*n* = 39, *W*(38) = 0.95, *p* = 0.06, *t*(114) = 6.30, *p* = 5.87 × 10^−9^, *d* = 1.47) for the anti-*E. coli* and 15.0 ± 9.2 (*n* = 36, *W*(35) = 0.89, *p* = 0.001, *t*(108) = 14.80, *p* = 9.86 × 10^−28^, *d* = 2.66) for the anti-*Enterococcus*, which are both significantly different from the blank measurements, but the anti-*Enterococcus* displayed the greatest effect size. Regarding the effect sizes, the anti-*Enterococcus* is found to be the best affinity binder.

Standard deviations for measurements with biotinylated bacteria revealed 52 ± 23% (*n* = 8, *W*(17) = 0.89, *p* = 0.24), which were significantly higher than without biotinylated bacteria, which showed values of 23 ± 11% (*n* = 16, *W*(15) = 0.88, *p* = 0.04; *chi-square* (1) = 10.53, *p* = 0.001, *t*(22) = 4.31, *p* = 2.82 × 10^−4^, *d* = 1.81). The measurements without biotinylated bacteria were used as blank measurements. Signals obtained are suspected to be unspecific bindings. On the other hand, the binding of living bacteria to the affinity binders seems to not be completely uniform, and a change in concentration, agglomeration of bacteria cells, living-to-dead cell ratio, or the steric hindrance of affinity binders by biotin on the cell surface could impact the measured CL signals.

Overall, the standard deviation for biotinylated *E. coli* was 42 ± 12% (*n* = 6) lower than that of biotinylated *E. faecalis,* 62 ± 28 (*n* = 6). One reason could be that the interactions are more preferable for gram-negative than for gram-positive bacteria due to the differences in the cell walls. Additionally, the biotinylation process was found to have a greater effect on *E. faecalis* regarding cell numbers and culturability, indicating that the cells were more influenced by this reaction. Nonetheless, an ANOVA revealed no significant difference (*F*(1,6) = 1.8, *p* = 0.22). For both bacteria, the respective antibodies worked best as affinity binders, which was expected, as commercial antibodies are designed to have a high affinity towards their antigen. PmB had a similar affinity for both bacteria, whereas lysozyme worked for *E. coli* as well but gave very unreproducible results for *E. faecalis*. 

Next, we checked if the previous used bacterial concentration of 1 × 10^8^ cells mL^−1^ would be suitable for this assay. For this, different concentrations of the biotinylated bacteria (1 × 10^7^, 5 × 10^7^ and 1 × 10^8^ cells mL^−1^) in PBS were measured. The measurements for 0 cells mL^−1^ (PBS) and 1 × 10^8^ cells mL^−1^ were the same as in the passage before, for which the results for the Shapiro–Wilk tests were also specified. For the following measurements, the significance level α for the post-hoc Conover tests was corrected to 0.008 using Bonferroni correction.

**Figure 4:**
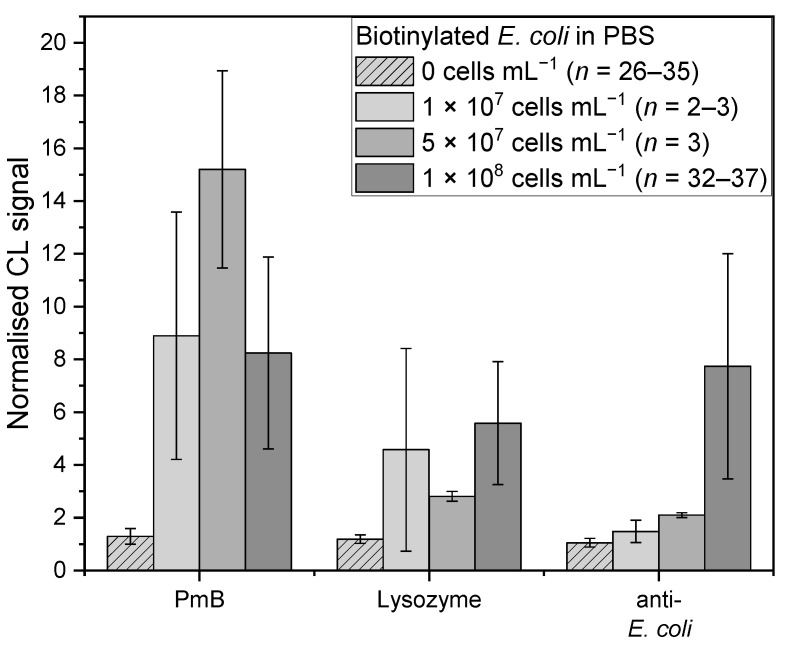
Measurements of biotinylated *E. coli* in PBS with different concentrations: 0 cells mL^−1^ (PBS, *n* = 26–35, lightest grey shaded), 1 × 10^7^ cells mL^−1^ (*n* = 2–3, lightest grey), 5 × 10^7^ cells mL^−1^ (*n* = 3, middle grey), and 1 × 10^8^ cells mL^−1^ (*n* = 32–37, darkest grey) for affinity binders PmB, lysozyme, and anti-*E. coli*, respectively.

For *E. coli*, the affinity binders PmB, lysozyme, and the anti-*E. coli* antibody were examined (Figure 4). For the anti-*E. coli* antibody (*chi-square* (3) = 59.41, *p* = 7.86 × 10^−13^), the highest mean-normalised CL signal was obtained for the highest concentration with a value of 7.7 ± 4.3 (*n* = 37), which showed no significant difference compared to 5 × 10^7^ cells mL^−1^ with 2.1 ± 0.1 (*n* = 3, *t*(74) = 2.49, *p* = 0.14). However, compared to the 1 × 10^7^ cells mL^−1^ with 1.5 ± 0.4 (*n* = 3, *t*(74) = 3.75, *p* = 3.48 × 10^−4^, *d* = 2.10), a significant difference was found. Between the two lowest concentrations, on the other hand, no significant difference was found (*t*(74) = 0.92, *p* = 0.36). The lowest concentration was the only one that showed no significant difference with the blank measurement (*t*(74) = 2.44, *p* = 0.02). 

For PmB (*chi-square* (3) = 58.08, *p* = 1.51 × 10^−12^), the highest mean-normalised CL signal was obtained for 5 × 10^7^ cells mL^−1^ (*n* = 3) with 15.2 ± 3.7. No significant difference was found compared to 1 × 10^7^ (8.9 ± 4.7, *n* = 3, *t*(73) = 1.92, *p* = 0.06) and 1 × 10^8^ cells mL^−1^ (8.2 ± 3.6, *n* = 36, *t*(73) = 2.62, *p* = 0.01). Additionally, these two concentrations were not significantly different (*t*(73) = 0.02, *p* = 0.99). For lysozyme (*chi-square* (3) = 46.57, *p* = 4.28 × 10^−10^), the mean-normalised CL signals for 1 × 10^7^ and 5 × 10^7^ cells mL^−1^ as well as 1 × 10^8^ cells mL^−1^ were in the same range, with 4.6 ± 3.8 (*n* = 2), 2.8 ± 0.2 (*n* = 3) and 5.6 ± 2.3 (*n* = 32), respectively. All three showed no significant difference (1 × 10^7^ and 5 × 10^7^: *t*(59) = 1.11, *p* = 0.27; 1 × 10^7^ and 1 × 10^8^: *t*(59) = 0.56, *p* = 0.58; 5 × 10^7^ and 1 × 10^8^: *t*(59) = 2.35, *p* = 0. 2). 

**Figure 5:**
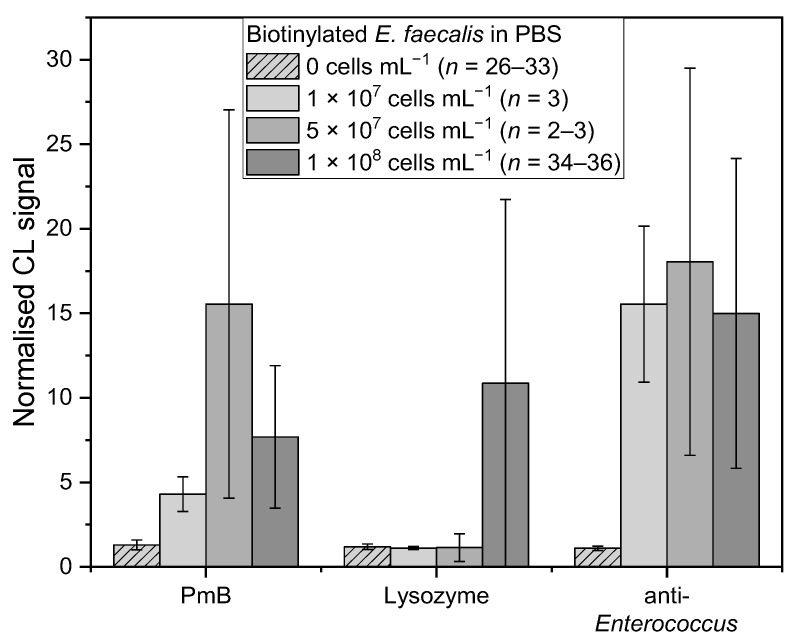
Measurements of biotinylated *E. faecalis* in PBS with different concentrations: 0 cells mL^−1^ (PBS, *n* = 26–33, lightest grey shaded), 1 × 10^7^ cells mL^−1^ (*n* = 3, lightest grey), 5 × 10^7^ cells mL^−1^ (*n* = 2–3, middle grey), and 1 × 10^8^ cells mL^−1^ (*n* = 34–36, darkest grey) for affinity binders PmB, lysozyme, and anti-*Enterococcus*, respectively.

For *E. faecalis,* the affinity binders PmB, lysozyme, and the anti-*Enterococcus* antibody were examined (Figure 5). PmB (*chi-square* (3) = 59.15, *p* = 8.92 × 10^−13^) showed a similar trend to *E. coli*, in which 5 × 10^7^ cells mL^−1^ induced the highest mean-normalised CL signal of 15.5 ± 11.5 (*n* = 3), whereas 1 × 10^7^ and 1 × 10^8^ cells mL^−1^ generated significant similar values of 4.3 ± 1.0 (*n* = 3, *t*(75) = 1.72, *p* = 0.09) and 7.7 ± 4.2 (*n* = 38, *t*(75) = 1.67, *p* = 0.10), respectively. These two values are significantly similar, too (*t*(75) = 2.48, *p* = 0.02). For lysozyme (*chi-square* (3) = 34.68, *p* = 1.42 × 10^−7^), mean-normalised CL signals for 1 × 10^7^ and 5 × 10^7^ cells mL^−1^ were 1.1 ± 0.1 (*n* = 3, *t*(61) = 0.87, *p* = 0.39) and 1.1 ± 0.8 (*n* = 2, *t*(61) = 0.02, *p* = 0.98), respectively, which are significant similar to the mean-normalised CL signals for blank measurements with PBS (*n* = 26). As mentioned before, for 1 × 10^8^ cells mL^−1^ a mean-normalised CL signal of 10.9 ± 10.9 (*n* = 34) is significantly different to the blank measurements and holds a very high standard deviation. For anti-*Enterococcus* (*chi-square* (3) = 54.70, *p* = 7.97 × 10^−12^), all three concentrations gave mean-normalised CL signals of the same range with 15.5 ± 4.6 (1 × 10^7^, *n* = 3), 18.0 ± 11.5 (5 × 10^7^, *n* = 3), and 15.0 ± 9.2 (1 × 10^8^, *n* = 36) (1 × 10^7^ and 5 × 10^7^: *t*(70) = 0.63, *p* = 0.53; 1 × 10^7^ and 1 × 10^8^: *t*(70) = 0.69, *p* = 0.50; 5 × 10^7^ and 1 × 10^8^: *t*(70) = 1.36, *p* = 0.19). According to the results for the anti-*E. coli* antibody with *E. coli* and the results for lysozyme with *E. faecalis*, a bacterial concentration of 1 × 10^8^ cells mL^−1^ is suitable for this screening assay.

### 3.4. Desorption Studies

For the investigation of the desorption properties of bacterial cells using the affinity binders, the microarray chips were eluted using six different desorption buffers in two different modes. The desorption buffer was either flushed over the chip or the chip was filled with it, shortly incubated, and then emptied. As a control, blank measurements were performed by measuring PBS and eluting the chip. A change in the normalised CL signal after desorption was observed here as well. One explanation would be the inactivation of bound HRP-streptavidin through peroxide or the desorption buffers. Therefore, obtained data for samples were displayed as the normalised residual CL signal.

(2) $$\mathrm{residual}\;\mathrm{CL}\;\mathrm{signal}=\frac{\mathrm{normalised}\;\mathrm{CL}\;\mathrm{signal}\;\left(\mathrm{after}\;\mathrm{elution}\right)}{\mathrm{normalised}\;\mathrm{CL}\;\mathrm{signal}\;\left(\mathrm{before}\;\mathrm{elution}\right)}$$

(3) $$\mathrm{normalised}\;\mathrm{residual}\;\mathrm{CL}\;\mathrm{signal}\;=\frac{\mathrm{residual}\;\mathrm{CL}\;\mathrm{signal}\;\left(\mathrm{sample}\right)}{\mathrm{mean}\;\mathrm{residual}\;\mathrm{CL}\;\mathrm{signal}\;\left(\mathrm{blank}\;\mathrm{measurement}\right)}$$

A value of 1 refers to a change of the normalised CL signal in the same range as for the blank measurements, any value below indicates a higher loss compared to the blank. The mean-normalised residual CL signal is given as the mean from the microarray chips measured with the corresponding standard deviation between experiments, whereas *n* is the number of experiments.

**Figure 6:**
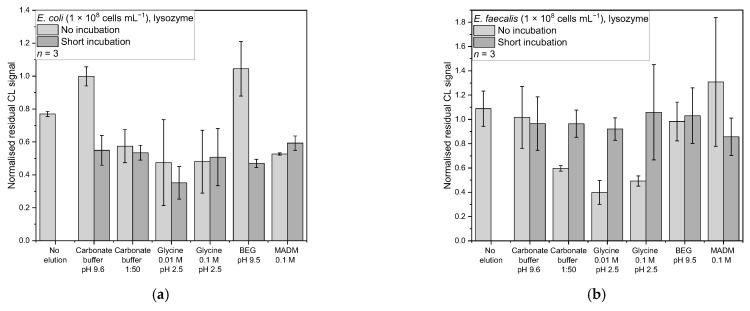
Normalised residual CL signals for desorption from the affinity binder lysozyme: (**a**) *E. coli* in PBS (*n* = 3, 1 × 10^8^ cells mL^−1^) (**b**) *E. faecalis* in PBS (*n* = 3, 1 × 10^8^ cells mL^−1^).

**Figure 7:**
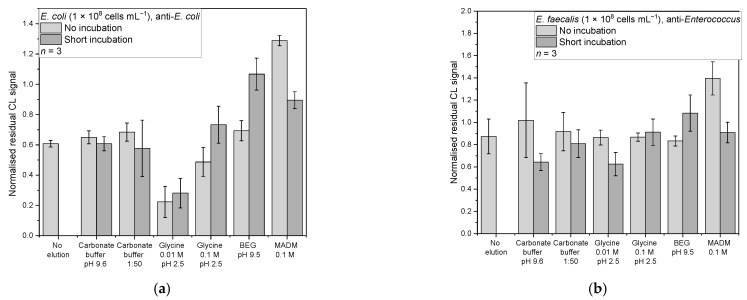
Normalised residual CL signals for desorption from the respective antibodies: (**a**) *E. coli* in PBS (*n* = 3, 1 × 10^8^ cells mL^−1^) from anti-*E. coli* antibody; (**b**) *E. faecalis* in PBS (*n* = 3, 1 × 10^8^ cells mL^−1^) from anti-*Enterococcus* antibody.

**Figure 8:**
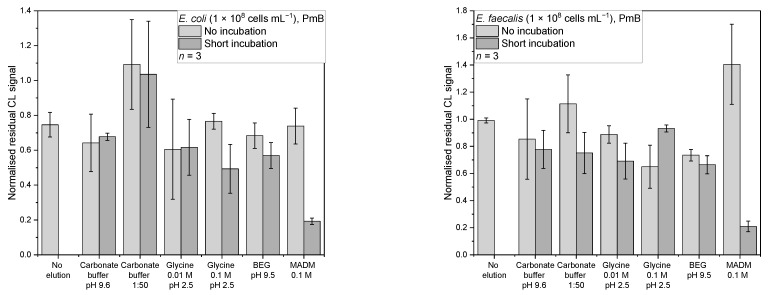
Normalised residual CL signals for desorption from the affinity binder PmB: (**a**) *E. coli* in PBS (*n* = 3, 1 × 10^8^ cells mL^−1^); (**b**) *E. faecalis* in PBS (*n* = 3, 1 × 10^8^ cells mL^−1^).

Based on the results from the concentration dependency, for the desorption studies, bacterial concentrations of 1 × 10^8^ cells mL^−1^ were used. For *E. coli*, the affinity binders PmB, lysozyme, and the anti-*E. coli* antibody were examined (all *n* = 3). The no-desorption controls showed a CL signal reduction for all three affinity binders, giving normalised residual CL signals ranging from 0.61–0.77 (Figure 6a, Figure 7 and Figure 8a). An unwanted desorption of bacterial cells through the CL reagents luminol and hydrogen peroxide could be the reason. Another cause could be a weak affinity leading to the cells being washed away in the washing step after taking the first picture, as here, the flow rate is higher than in the other washing steps. 

For *E. faecalis*, the affinity binders PmB, lysozyme, and the anti-*Enterococcus* antibody were further examined for their desorption properties (all *n* = 3). The no-elution control for the first two showed mean-normalised CL signals of 0.99 ± 0.11 and 1.09 ± 0.19, respectively, indicating a good affinity of the affinity binders for the bacteria (Figure 6b and Figure 7b). Only for the antibody, a mean-normalised CL signal decrease of 0.87 ± 0.10 was observed (Figure 8b). 

#### 3.4.1. Lysozyme

For *E. coli* and lysozyme, regarding the mean-normalised residual CL signal, the best desorption strategy was found to be 0.01 M glycine at pH 2.5 incubated for 1 min with a mean-normalised residual CL signal of 0.35 ± 0.18 (Figure 6a). Other desorption strategies were in the range of 0.47–0.59, except for carbonate buffer (pH 9.6) and the BEG buffer (pH 9.5) applied without incubation, for which the mean-normalised residual CL signals were 1.00 ± 0.19 and 1.04 ± 0.12, respectively. 

The Kruskal–Wallis test showed that there is a significant difference between the data (*chi-square* (12) = 26.38, *p* = 0.01). The following Conover test (corrected α = 6.41 × 10^−4^) revealed that the result from 0.01 M glycine with short incubation significantly differs from the no-elution control (*t*(26) = 4.45, *p* = 1.45 × 10^−4^, *d* = 3.39), carbonate buffer without incubation (*t*(26) = 5.20, *p* = 2.01 × 10^−5^, *d* = 5.23), and BEG without incubation (*t*(26) = 5.14, *p* = 2.31 × 10^−5^, *d* = 5.61). At the same time, it was the only one significantly different from the no-elution control. The Cohen’s *d* values are all in the range for great effects. Concluding this, the 0.01 M glycine with incubation is the buffer of choice, although it is not significantly different from most other elution strategies.

For *E. faecalis* and lysozyme (Figure 6b), the best desorption buffer according to the mean-normalised residual CL signal was the 0.01 M glycine (pH 2.5) without incubation, with a normalised residual CL signal of 0.40 ± 0.10. The other buffers yielded normalised residual signals between 0.49 and 1.31. The Kruskal–Wallis test showed a significant difference in between the data (*chi-square* (12) = 23.52, *p* = 0.02). The following Conover test (corrected α = 6.41 × 10^−4^) revealed, that the result from 0.01 M glycine without incubation only significant differs from the no elution control (*t*(26) = 3.93, *p* = 5.54 × 10^−4^, *d* = 2.97) and MADM without incubation (*t*(26) = 3.91, *p* = 5.54 × 10^−4^, *d* = 5.23). Again, it was the only one significantly different from the no-elution control, and the Cohen’s *d* values are in the range for great effects.

#### 3.4.2. Antibodies

For the *E. coli* and the anti-*E. coli* antibody, (Figure 7a) 0.01 M glycine at pH 2.5 gave the lowest mean-normalised residual CL signals of 0.22 ± 0.10 without incubation and 0.28 ± 0.10 with short incubation. The other desorption methods showed mean-normalised residual CL signals from 0.49–1.29. An ANOVA showed that there are significant differences between the data (*F*(12,26) = 8.36, *p* = 3.5 × 10^−6^). The post-hoc test showed that these methods are the only ones that differ from the no-elution control (no incubation: *p* = 0.001, *d* = 4.24; incubation: *p* = 0.08, *d* = 3.60). They both are significant similar (*p* = 1.00), which indicates that a short incubation does not enhance the elution. The only other elution mode they do not differ from is the 0.1 M glycine without incubation (no incubation: *p* = 0.06; incubation: *p* = 0.27). The other elution modes are all significantly different from the two glycine elution modes (*p*-values all below 0.05). The effect sizes lie between 3.22 and 5.62, indicating great effects. Glycine buffers are widely used for the desorption of antibodies in affinity chromatography, so desorption was expected. 

For the *E. faecalis* and the anti-*Enterococcus* antibodies, carbonate buffer and the 0.1 M glycine with short incubation had the best mean-normalised residual CL signals of 0.64 ± 0.08 and 0.63 ± 0.11, respectively (Figure 7b). The other buffers had values between 0.81 and 1.40. A Kruskal–Wallis test showed that there is a significant difference in between the data (*chi-square* (12) = 26.38, *p* = 0.04). The following Conover test (corrected α = 6.41 × 10^−4^) revealed that the only significant difference was found between MADM without incubation (1.40 ± 0.15) with the carbonate buffer (*t*(26) = 4.48, *p* = 1.32 × 10^−4^, *d* = 5.14) and the glycine (*t*(26) = 4.43, *p* = 1.48 × 10^−4^, *d* = 5.26), both with a short incubation. However, there was no significant change from the no-elution control compared to all of the elution modes. This finding suggests that the affinity between *E. faecalis* and its corresponding antibody could not be broken by the used desorption strategies of different pH values and protein/salt contents.

#### 3.4.3. PmB

For PmB and *E. coli* (Figure 8a), most of the desorption strategies showed similar mean-normalised residual CL signals between 0.49–0.77 except for the 1:50 dilution of carbonate buffer, which showed without and with incubation values of 1.09 ± 0.26 and 1.03 ± 0.30, respectively. But the lowest mean-normalised residual CL signal of 0.19 ± 0.02 was found for 0.1 M MADM in combination with the short incubation. 

An ANOVA showed that there are significant differences between the data (*F*(12,26) = 5.75, *p* = 9.55 × 10^−5^). The post-hoc test showed, that MADM with incubation is the only one significantly different from the no elution control (*p* = 0.01, *d* = 3.41). It also is significantly different from carbonate buffer with incubation (*p* = 0.047, *d* = 2.98), carbonate buffer 1:50 (no incubation: *p* = 2.18 × 10^−5^, *d* = 5.54; incubation: *p* = 6.24 × 10^−5^, *d* = 5.19), 0.1 M glycine without incubation (*p* = 0.01, *d* = 3.53), and BEG without incubation (*p* = 0.04, *d* = 3.02). Additionally, MADM without incubation was significantly different (*p* = 0.02, *d* = 3.36), indicating that a short incubation step is necessary for successful elution. 

Initially, MADM should have been used for the desorption from ConA, but because of the multiplexing approach of this screening chip and the simultaneous test for other affinity binders, this unexpected result was obtained. A literature search revealed that bacterial cell wall lectins are known to have an affinity towards sugars [39]. Affinity between MADM and *E. coli* seems to be stronger than between *E. coli* and PmB.

For PmB and *E. faecalis* (Figure 8b), most of the desorption strategies gave a mean-normalised residual CL signal range of 0.65–1.11. Again, the 0.1 M MADM with short incubation showed the lowest mean-normalised residual CL signal of 0.21 ± 0.04. A Kruskal–Wallis test showed that there is a significant difference between the data (*chi-square* (12) = 29.78, *p* = 0.003). The following Conover test (corrected α = 6.41 × 10^−4^) showed that not only was the MADM with incubation significantly different from the no elution control (*t*(26) = 5.73, *p* = 4.94 × 10^−6^, *d* = 4.99) but that the 0.1 M glycine without incubation (*t*(26) = 4.01, *p* = 4.53 × 10^−4^, *d* = 2.18) and BEG with incubation (*t*(26) = 4.13, *p* = 3.25 × 10^−4^, *d* = 2.09) were as well. Focusing on MADM, an significant difference to the no incubation mode was observed (*t*(26) = 6.75, *p* = 3.66 × 10^−7^, *d* = 7.63). Here, the effect an incubation step can have is very obvious. MADM with incubation is also significantly different from carbonate buffer 1:50 without incubation (*t*(26) = 5.73, *p* = 4.94 × 10^−6^, *d* = 5.77), 0.01 M glycine without incubation (*t*(26) = 4.39, *p* = 1.66 × 10^−4^, *d* = 4.33), and 0.1 M glycine with incubation (*t*(26) = 5.03, *p* = 3.10 × 10^−5^, *d* = 4.61).

## 4. Conclusions

In this work, a flow-based CL microarray was developed for the rapid screening of affinity binders for the capture of bacteria. Both gram-positive and gram-negative bacteria were successfully biotinylated for their detection via HRP-streptavidin and CL. The four affinity binders PmB, lysozyme, anti-*E. coli* antibody, and anti-*Enterococcus* antibody were immobilised on the microarray surface and screened using this assay. For *E. coli*, the respective antibody was found to be the best affinity binder, followed by PmB, and these were best eluted with 0.01 M glycine and MADM, respectively. For *E. faecalis,* the respective antibody was found to be the best affinity binder, although in this study, no suitable desorption method was found. For both bacteria, the elution from PmB with MADM could be enhanced by a short incubation step. The necessity of such a screening platform to simplify the search for new combinations of affinity binders and bacteria was shown, as desorption behaviours differed sometimes between *E. faecalis* and *E. coli*. One important advantage of the screening platform was found to be the ability of testing desorption buffers on the whole microarray chip at once, leading in our case to unexpected new desorption reagents. 

With this study, the principle of a microarray-based affinity binder screening platform was established using CL as detection method, but the principle could also be applied to microarray assays using fluorescence-based or label-free detection. The microarray has space for up to 18 × 5 different affinity binders, which enables a high throughput in screening. Desorption buffers used in this work were only a selection of buffers that could be screened for desorption. Additionally, the methods for desorption can be expanded as needed—for example, through longer incubation intervals. After successful screening of affinity binders and respective desorption methods, they can be applied for affinity enrichment of bacteria—for example, affinity-based filtration. 

## Figures and Tables

**Table 1:** Measuring program on the MCR-R.

Step	Process	Volume/µL	Flow Rate/µL s^−1^	Comments
1	Transport sample to chip	118	50	
2	Sample incubation	600	1	10 increments, pause 30 s
3	Wash chip	2000	150	
4	Block chip	90	50	Casein in PBS
		600	5	
5	Wash chip	2000	150	
6	Incubate HRP-streptavidin	118	50	
		600	2	
7	Wash chip	2000	150	
8	Add CL reagents	400 (200 each)	100	Luminol and hydrogenperoxide
9	Take image			60 s exposure
10	Flush chip	1000	200	
11	Remove chip			Manual desorption
12	Flush device	2500	500	Sample loop
		2500	500	Sample way
		2500	500	Chip (extra washing chip)
13	Insert chip			
14	Incubate HRP-streptavidin	118	50	
		600	2	
15	Wash chip	2000	150	
16	Add CL reagents	400 (200 each)	100	Luminol and hydrogenperoxide
17	Take image			60 s exposure
18	Flush device	2500	500	Sample loop
		2500	500	Sample way
		2500	500	Chip

## Data Availability

Data will be made available upon reasonable request.

## Supplementary Materials

The following supporting information can be downloaded at: https://www.mdpi.com/article/10.3390/s22228606/s1; Table S1: Pathway for reagents during the measuring program on the MCR-R., Figure S1: Schematic fluidic plan of the MCR-R.
