# Supplementary material for: Flow-Based Chemiluminescence Microarrays as Screening Platform for Affinity Binders to Capture and Elute Bacteria
Source: Sensors (Basel). 2022 Nov 8;22(22):8606. doi: 10.3390/s22228606 (PMC9693076; doi:10.3390/s22228606)
Supplement: Supplementary file 1 [file sensors-22-08606-s001.zip › sensors-1976495-supplementary.pdf]

Supplementary information

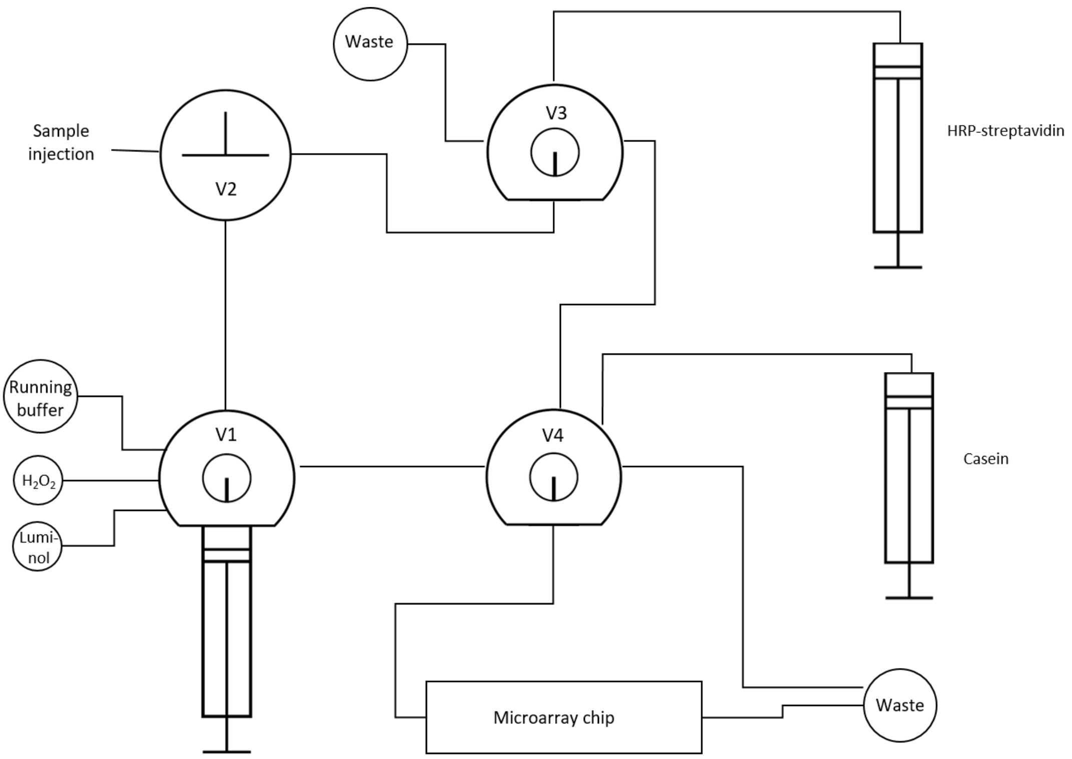

Figure S1: Schematic fluidic plan of the MCR-R. The positions and lengths of the tubing is just for demonstration purposes.

Table S1: Pathway for reagents during the measuring program on the MCR-R.

Process	Pathway
Sample	Running buffer – V1 – V2– V3 – V4 – Chip – Waste
Wash chip	Running buffer – V1 – V2– V3 – V4 – Chip – Waste
Blocking	Casein – V4 – Chip – Waste
HRP-streptavidin	HRP-streptavidin – V3 – V4 – Chip – Waste
CL-reagents	Hydrogen peroxide – V1 + Luminol – V1 – V4 – Chip – Waste
Flush device "Sample way"	Running buffer – V1 – V2 – V3 – V4 – Waste
Flush device "Sample loop"	Running buffer – V1 – V2 – V3 – Waste
Flush device "Chip"	Running buffer – V1 – V4 – Chip – Waste
